# Identifying communication-related predictors of patient satisfaction in a briefing prior to contrast-enhanced computed tomography

**DOI:** 10.1186/s13244-019-0778-7

**Published:** 2019-09-23

**Authors:** Valentina Scholz, Sandra Lange, Britta Rosenberg, Marie-Luise Kromrey, Annika Syperek, Norbert Hosten, Thomas Kohlmann, Michael Kirsch

**Affiliations:** 1 Department of Diagnostic Radiology and Neuroradiology Greifswald, Ferdinand-Sauerbruch-Straße, 17475 Greifswald, Germany; 2 Section Methods in Community Medicine, Walther-Rathenau-Straße 48, Institute for Community Medicine, 17475 Greifswald, Germany

**Keywords:** Doctor-patient communication, Informed consent, Patients’ satisfaction, Contrast-enhancement

## Abstract

**Background:**

This study aimed to prospectively investigate patients’ satisfaction with briefings before computed tomography (CT) examinations, determine feasibility, and identify factors influencing patient satisfaction independent of patient and physician characteristics.

**Methods:**

One hundred sixty patients received information by a radiologist prior to contrast-enhanced CT examinations in an open, prospective, two-center, cross-sectional study (including the introduction of the radiologist, procedure, radiation exposure, possible side effects, and alternatives). Afterwards, patients and radiologists evaluated the briefing using a standardized questionnaire. Additionally, factors such as age, socioeconomic status, inpatient/outpatient status, length of the radiologist’s professional experience, duration of the briefing, clarity of the radiologist’s explanations as perceived by patients, and the duration of communication were obtained in this questionnaire. Subsequently, three classes of influencing factors were defined and entered stepwise into a hierarchical regression.

**Results:**

Patient satisfaction ratings differed significantly by type of hospitalization, perceived type of communication, and patient gender. Hierarchical regression analysis revealed that perceived clarity was the strongest predictor of patients’ satisfaction when controlling for the patient and physician characteristics.

**Conclusions:**

Patients appeared to be satisfied with the briefing prior to CT examination. The mean briefing time (2 min 35 s) seemed feasible. Patients’ demographics influenced satisfaction. To improve patients’ satisfaction with briefings before contrast-enhanced CT, radiologists should aim to clarify their communication.

## Key points

- Patient’s characteristics (e.g., age, gender, education) considerably influence their satisfaction with briefings before contrast-enhanced computed tomography but cannot be actively affected by the radiologist to improve communication.
- Physician’s characteristics (e.g., work experience) are of minor importance for patient satisfaction.
- When controlling for the patient and physician characteristics, perceived clarity of the communication situation is the strongest predictor of patient satisfaction with briefings before contrast-enhanced computed tomography.
- The findings of this study support the possible shift of the briefings’ tasks to physician’s assistants.

## Background

In effective doctor-patient communication, patients expect their doctors to listen to them, maintain eye contact, and take an appropriate amount of time out of their schedules for conversations [1]. Radiologists generally have the opportunity to fulfill these expectations in two situations: they can talk to patients about the planned procedure during a briefing before the examination, and they can present and explain the results to them afterwards, for instance, on their workstation [2].

These two distinct conversational settings come with different circumstances. Presenting results immediately after the examination requires work experience [2]. The content of a pre-examination briefing, on the other hand, is relatively well defined and does not differ substantially from patient to patient. Thus, it even seems conceivable that a radiology physician’s assistant may lead this type of conversation with a patient.

Previous research has shown that when a physician meets the patient’s expectations, the patient is more satisfied [3]. Ultimately, several studies have shown that the recovery process is influenced by patient satisfaction [4–15].

Also, patients need for medical information increases [16, 17] but unfortunately, radiologists do not meet those needs and seem to perceive themselves primarily as the “doctor’s doctor” [18] rather than their patients’ doctors. Thus far, this perception has led to a lack of research on patient satisfaction in radiology compared to other specialties, who put more weight on doctor-patient communication, as seen in telemedicine (e.g., neurological stroke diagnosis). Relevant research has been conducted in the fields of orthopedics and anesthesiology [10, 12, 19]. Radiologists have comparable expertise for the field of mammography, which they could transfer to other examinations [20, 21]. It is no coincidence that the radiological subspecialty with the most results concerning doctor-patient communication is mammography. Traditionally, mammography is the radiological field with the most intense doctor-patient interactions. However, most (but not all: [22–24]) studies of mammography discuss the communication of examination results rather than a briefing taking place before mammography.

In the present study, we aimed to identify predictors of patient satisfaction that were independent of physician and patient characteristics.

## Materials and methods

### Study design

We studied briefings before contrast-enhanced computed tomography (CT) for two reasons: CT is a widely performed examination, and its radiation exposure is among the higher of radiological exams. Information about the latter is a crucial point of radiological briefings, in particular after Council Directive 59/13 was introduced [25, 26]. The study was conceived by one of the authors, a social scientist (T.K.), as an open, prospective, two-center, cross-sectional study in a university hospital and a private radiology practice. The sample size was based on common experience with studies of this type. The study cohort included 160 patients (80 men, 80 women, mean age 61.5 ± 13.3 years, age range 23–84 years) who took part in a briefing prior to contrast-enhanced CT over the course of 3 months; 16 radiologists (five female and 11 male, 1 to 20 years of professional experience) performed the briefings. The local ethics committee approved the study and written informed consent to participate in this study was obtained from all voluntarily participating patients. The local physicians’ staff association was informed about the study, and the participating radiologists also consented.

### Inclusion/exclusion criteria

Inclusion criteria included

- Legal age for consent (≥ 18 years of age)
- Karnofsky Performance Status Scale > 40%
- Providing informed consent

We aimed to achieve an even sex ratio, with 80 female patients (50%) and 80 male patients (50%). This was achieved by including only men after a total of 80 women had been enrolled. There were no restrictions regarding comorbidities, medication usage, or other clinical parameters. We aimed at the consecutiveness of patients, but not all patients could take part. Patients not able to participate were mostly those referred from the intensive care units and emergency rooms.

During the survey period, 289 patients underwent a CT examination. Patients were excluded from the study (*n* = 129) if their legal competence was questionable or unclear. There were no other reasons for exclusion.

### Pre-examination briefing procedure

Initially, the radiologist presented him- or herself formally to the patient. The briefing then included the legally defined items: the establishment of the procedure and its purpose, explanation of radiation exposure, solicitation of information and communication about adverse reactions including thyrotoxic crisis and contrast agent-induced nephropathy, and alternatives to the procedure. Development of cancer as a possible result of radiation exposure was mentioned as well as induction of cataract. Among adverse events, skin, soft-tissue, and nerve damage were communicated as possible sequelae of contrast medium injection. The possibility of hypersensitivity reactions to contrast medium was explained and life-threatening outcomes as swelling of the larynx, breathing impairment, cardiac failure, and seizures were described. Minor side effects like nausea and vomiting or panic attacks were also mentioned. Each radiologist chose the way of explaining these possible complications by him- or herself.

Sixteen radiologists with a mean work experience of 7.4 years gave the briefings. A study nurse measured the actual duration of each briefing.

### Content of the questionnaires/parameters evaluated

Two questionnaires were designed to assess patients’ and physicians’ perceptions of the briefing. The questionnaires were created specifically for this study, and the patients’ questionnaire relied on other studies that previously measured patient satisfaction [27–29]. For the physician questionnaire, studies such as one on communicating results of mammography [20] were used. Questionnaires contained open-ended questions, closed multiple-choice questions with preformulated answers, and Likert-type 6-point answer scales for the assessment of target criteria. Even-numbered scales were used to prevent a tendency toward the middle. Gender and age of patients were assessed, and work experience (in years) of the physicians was assessed. Independent variables also included the perceived clarity of the briefing, perceived necessity of the briefing, duration of the briefing as perceived by the patient, physician’s satisfaction, and patients’ satisfaction perceived by the physician. Satisfaction perceived by the patient was the only dependent variable. Patients’ satisfaction was assessed on a Likert-type 6-point answer scale (“1” = “not at all satisfied” to “6” = “fully satisfied”). The actual duration of the talk as measured by the study nurse was another independent variable.

In accordance with approaches of previous studies ([30, 31]), we determined a briefing to be patient-centered from the number of positive answers to the following questions: “Was there enough time for understanding the doctor’s explanations?”; “Did you understand the briefing?”; “Could you ask questions?”; and “Do you remember the doctor’s name from his introduction?” We, therefore, measured patient-centeredness by doctors’ verbal behavior [30]. If answers to at least three of these questions were “yes,” we considered the briefing to be patient-centered.

As mentioned by the European Society of Radiology (ESR), radiologists should be able to communicate clearly [32]. Thus, in this study, patients’ perception of clarity was examined independently from patient-centeredness on a Likert-type-6-point scale.

#### Patient questionnaire

The patient questionnaire focused on the evaluation of patient satisfaction. Patients went through it with guidance and assistance from the study nurse when necessary. Patients were assured about the confidentiality of their answers. The contents of the patient questionnaire were unknown to the radiologists leading the briefing. The first part included sociodemographic questions (e.g., age, gender, education), questions about inpatient/outpatient status, and specific patient-related questions about the medical briefing (e.g., “Have you previously been briefed about an examination involving X-rays?”, “Did you use sources of information about contrast-enhanced computed tomography?”)

The second part contained specific questions about the patient’s perception of the medical briefing (e.g., “How long did the briefing last?”, “How satisfied were you with the informed consent briefing?”) as well as statements about the medical briefing which the patient could agree or disagree with (e.g., “An informed consent briefing is necessary.”, “The informed consent briefing was clear.”).

#### Physician questionnaire

The physician questionnaire focused on the physician’s satisfaction with the briefing and the information supplied to the patient. The questionnaire included general questions about the physician’s work experience (e.g., “How long have you been working in radiology?”). The second part assessed information about the medical briefing. The physician was asked to evaluate his/her satisfaction with the medical briefing in general (e.g., “How satisfied were you with the informed consent briefing?”), his/her satisfaction with specific aspects of the briefing (e.g., “My verbalization was appropriate”) as well as the perceived type of communication (patient-centered, physician-centered, equal)*.* The physician was also asked to assess his/her perception of the patients’ satisfaction to evaluate accordance with the physicians’ perception of his/her satisfaction. Furthermore, the questionnaire evaluated specific information about the consent of the briefing (e.g., “The informed consent briefing included information about (tick one or more): (1) diagnosis (2) side effects (3) cause of disease (4) therapy (5) course of disease (6) recommendations (7) other”). Due to privacy policies, the questionnaire did not include any questions regarding sociodemographic data.

A study nurse used both questionnaires (patient and physician) for a structured interview immediately after the briefing procedure.

### Statistical analysis

Statistical analyses were performed using SPSS for Mac, Version 25.0 (Armonk, NY: IBM Corp).

For descriptive analysis, we calculated means and standard deviations for patients’ satisfaction ratings. Differences in the distributions of categorical variables were analyzed by the Chi-square test*.* Variables that used Likert-type scale responses (e.g., satisfaction, clarity) were treated as continuous data according to Harpe [33]. For this purpose, Likert-type scales provided verbal anchors for extremes as well as numerical labels for each option. Distances between options were distributed equally.

An independent samples Student *t* test was used to analyze differences in patients’ satisfaction by dichotomous categorical variables (e.g., gender, type of communication). For non-dichotomous categorical variables (education and age categorized in groups), one-way ANOVA was used to analyze between-group differences.

Physicians’ perceptions of their satisfaction were compared to physicians’ perceptions of patients’ satisfaction and patients’ perceptions of their satisfaction using a dependent Student *t* test.

Effect sizes were calculated to evaluate the clinical significance of statistically significant differences.

Hierarchical regression was used to determine whether communication variables (e.g., perceived clarity and perceived duration) were significant predictors of patient satisfaction when controlling for the patient (e.g., age, gender, education) and physician (e.g., work experience) characteristics. Age, work experience, and perceived and measured duration of communication were entered as continuous variables into the regression model. Categorical variables were dummy coded. The non-dichotomous categorical variable “education” was dummy coded into higher education “yes” (> 10 years of education) vs. “no” (≤ 10 years of education).

All statistical tests were two-tailed, with a significance level of 5%.

## Results

### Demographics

Patient demographics and attributes are presented in Table 1. Most subjects had left secondary school after 10 years of education, and only a minority had passed a matriculation examination or received a college-equivalent education. There were slightly fewer inpatients than outpatients in the study group.

**Table 1:** Descriptive data and statistical analysis of categorical variables

Variable	Category	N (%)	ϰ^2^
Gender	Female	80 (50.0)	0.000
	Male	80 (50.0)	
Higher education^a^	No	122 (76.3%)	44.100****
	Yes	38 (23.8%)	
Hospitalization	Inpatient	71 (44.4)	2.025
	Outpatient	89 (55.6)	
Prior informed consent briefing	Yes	123 (76.9)	46.225****
	No	37 (23.1)	
Supplemental source(s) of information	Yes	44 (27.5)	32.400****
	No	116 (72.5)	
Perceived type of communication^b^	physician-centered	82 (51.2)	0.100
	patient-centered	78 (48.8)	

*N* number of cases

****p < 0.0001

^a^Higher education “Yes” > 10 years of education, higher education “No” ≤ 10 years of education

^b^Perceived by patient

Physicians had an average of 8.17 ± 7.98 years of professional experience (median 5 years, range 0–20 years).

### Briefing

While 76.9% of patients had already experienced a prior CT examination and the associated briefing, the procedure was new to 23.1% of patients (Table 1). A total of 72.5% (*n* = 116) of all patients had not consulted other informational material (patient brochures, websites, television programs) prior to the examination.

### Duration and type of communication

Briefing patients prior to contrast CT examination took 155 s on average (range 60–780 s, SD 155 s). The mean perceived duration of the briefing, however, was longer at 344 s (range 60–1200 s, SD 343 s).

Radiologists perceived the communication to be patient-centered in 7.5% of briefings and physician-centered in 57.5%. In 35% of all briefings, radiologists perceived the patient’s and their own shares of the briefing to be equal. Patients perceived 48.8% of briefings as patient-centered and 51.2% as physician-centered.

### Patient satisfaction

Overall satisfaction with the briefing perceived by patients was high (Mean = 5.1, *SD* = 1.042). Physicians estimated patients’ satisfaction (Mean = 5.32, *SD* = 0.954) as high as their own satisfaction with the briefing (Mean = 5.36, *SD* = 0.827) but overestimated patients’ actual perception of the briefing (*t* (159) = 2.868, *p* = 0.005, *r* = 0.22).

Patients’ satisfaction differed significantly (*t* (158) = − 5.241, *p* < 0.0001, *r* = 0.38) between inpatients (Mean = 4.7, *SD* = 1.085) and outpatients (Mean = 5.5, *SD* = 0.790). Patients who perceived a patient-centered (Mean = 5.5, *SD* = 0.849) type of communication were also significantly more satisfied (*t* (158) = − 4.898, *p* < 0.0001, *r* = 0.36) than patients who perceived a more physician-centered (Mean = 4.7, *SD* = 1.080) type of communication. Gender was also significantly associated with satisfaction (*t* (158) = − 2.306, *p* = 0.022, *r* = 0.18). Women were more satisfied (Mean = 5.3, *SD* = 1.043) than men (Mean = 4.9, *SD* = 1.014). There was no significant difference in satisfaction according to other patient-related variables, such as the use of supplemental sources of information, education, or prior informed consent briefing (see Table 2).

**Table 2:** Statistical analysis of patient satisfaction

Variable	Category	Mean (SD)	*t* value	Effect size (*r*)^a^
Gender	Female	5.3 (1.043)		
	Male	4.9 (1.014)	− 2.306*	0.18
Higher education^b^	No	5.13 (.995)		
	Yes	4.95 (1.184)	0.949	0.08
Hospitalization	Inpatient	4.7 (1.085)		
	Outpatient	5.5 (.790)	− 5.241****	0.38
Prior informed consent briefing	Yes	5.06 (1.058)		
	No	5.19 (.995)	0.676	0.05
Supplemental source(s) of information	Yes	4.91 (1.117)		
	No	5.16 (1.010)	1.337	0.11
Perceived type of communication^c^	Physician-centered	5.5 (.849)		
	Patient-centered	4.7 (1.080)	− 4.898****	0.36

*p < 0.05; ****p < 0.0001

^a^According to Cohen [34] *r* > 0.10 indicated a small effect, *r* > 0.30 indicated a medium effect, and *r* > 0.50 indicated a large effect

^b^Higher education “Yes” > 10 years of education, higher education “No” ≤ 10 years of education

^c^Perceived by patient

### Hierarchical regression analysis

We used hierarchical regression analysis to examine whether communication-related variables such as perceived clarity and duration would significantly predict patients’ satisfaction if the patient (e.g., age, gender, education) and physician (work experience, physicians’ satisfaction, physicians’ perception of patient satisfaction) characteristics were controlled. Therefore, we entered the characteristics of patients, physicians and briefings stepwise into the regression model, and analyzed changes in the R-square value.

As shown in Table 3, patient variables significantly predicted patients’ satisfaction by explaining 20% of the variance (*F* (6,153) = 6.425, *p* < 0.0001), with inpatient/outpatient status being the strongest predictor (*β* = 0.400, *p* < 0.0001). Physician characteristics explained less than 1% of the variance in patient satisfaction (Δ*F* (3150) = 0.517, *p* = 0.671). In contrast, communication variables explained an additional 8% of variance (Δ*F* (5145) = 3.117, *p* = 0.011). In model 3, perceived clarity was the strongest predictor of patients’ satisfaction (*β* = 0.249, *p* = 0.002). Patient-centered communication, perceived necessity, and the measured duration of the talk did not significantly predict patients’ satisfaction with the pre-examination briefing (see Table 3).

**Table 3:** Hierarchical regressions for the prediction of patients’ satisfaction

	Predictor	Standardized β coefficient	*R* ^2^	Δ*F* test
Model 1: patient variables	Age (years)	− 0.041		
	Gender (Male)	0.145		
	Supplemental source(s) of information	− 0.079		
	Prior informed content briefing	− 0.033		
	Hospitalization (Inpatient)	0.400****		
	Higher education^a^	− 0.133	0.201	6.425****
Model 2: patient and physician variables	Age (years)	− 0.051		
	Gender (Male)	0.147		
	Supplemental source(s) of information	− 0.083		
	Prior informed content briefing	− 0.029		
	Hospitalization (Inpatient)	0.352*		
	Higher education^a^	− 0.125		
	Physicians’ work experience (years)	0.010		
	Physicians’ satisfaction (Likert scale 1–6)	− 0.005		
	Physicians’ perception of patients’ satisfaction (Likert scale 1–6)	0.100	0.209	0.517
Model 3: patient, physician, and communication variables	Age (years)	− 0.019		
	Gender (Male)	0.141		
	Supplemental source(s) of information	− 0.053		
	Prior informed content briefing	− 0.021		
	Hospitalization (Inpatient)	0.182		
	Higher education^a^	− 0.134		
	Physicians’ work experience (years)	0.090		
	Physicians’ satisfaction (Likert scale 1–6)	− 0.013		
	Physicians’ perception of patients’ satisfaction (Likert scale 1–6)	0.084		
	Necessity^b^ (Likert-Scale 1–6)	− 0.081		
	Clarity^b^ (Likert scale 1–6)	0.227**		
	Measured duration (seconds)	0.085		
	Perceived Duration^b^ (s)	− 0.007		
	Patient-centered communication^b^	0.165	0.286	3.117*

Unless otherwise stated, variables were entered dummy coded with 0 = “No” and 1 = “Yes”. Degrees of freedom for the *F* test corresponding to model 1 are (6153), corresponding to model 2 are (3150), and corresponding to model 3 are (5145)

*p < 0.05; **p < 0.01; ****p < 0.0001

^a^Higher education “Yes” > 10 years of education, higher education “No” ≤ 10 years of education

^b^Perceived by patient

## Discussion

An essential issue of researching radiology’s role in current health systems that merits attention is the investigation of patients’ needs and expectations [18]. The individual situations of patients are often not taken into consideration in the field of diagnostic radiology. This could have a negative impact on patient satisfaction and treatment results [4–15]. Consequently, patient-oriented care should receive the same level of attention as evidence-based practices [35].

Effective communication between physicians and patients should be perceived as a central component not only of treatment-oriented medical specialties but also of diagnostic specialties, i.e., radiological patient care. It was established in the move away from a patronizing medicine in the 1970s to 1990s, mainly in oncology and other specialties [5, 14, 36]. Radiological research covering this topic is still sparse. As mentioned before, the communication may take place prior to the exam, in a briefing or in an informed consent conversation, or afterwards when demonstrating results to the patient. While the second situation has been the subject of radiological research [2, 20], little attention has been paid to the communication prior to an examination thus far. In such a briefing, the patients should not only receive insights on the causes and consequences of their disease or condition but also on the risks and side effects of medical procedures, according to the new European Directive 59/2013 [25, 26]. In an ideal setting, this communication should set the foundation for a trusting patient-doctor relationship.

The communication between radiologists and their patients helps the physician understand the patient’s expectations and opinions [5, 37, 38]. Increased satisfaction with this briefing can positively influence the course and outcome of medical treatments [4–15]. Several studies show that patient satisfaction is correlated with the length of stay, the number of follow-up visits, and resulting costs [8, 31].

“Satisfaction” is not a universally defined term but rather a complex construct with little conceptual consistency [39]. To overcome the lack of conceptual consistency, evaluation of patient satisfaction with health care is a conceptional complex research field [40]. “Patient satisfaction” is a multidimensional construct influenced by several factors, such as conversational setting (i.e., the invested time, place, etc.), appreciation of the patient’s worries and fears, and comprehensibility of the briefing; research should improve our understanding of these points.

Some content of briefings prior to contrast-enhanced CT examinations is indeed consented already. Briefings usually include information on possible adverse reactions to contrast agents and the possible effects of radiation exposure. Fixed content of the briefing makes it easier to delegate this conversation to a physician’s assistant.

### Time necessary for informed consent briefing

The mean briefing time of 155 s in our study was more prolonged than in a study by Langewitz et al. [41], who observed that patients who were greeted by an open question by their treating physician spoke for a mean duration of approximately 90 s. Even so, the time span is such that it could be integrated into a radiological schedule. Our results further show that patients tended to overestimate both the duration of the briefing and the time they talked to their doctor.

### Satisfaction with informed consent briefings

The overall mean satisfaction of patients was high at 5.1. Radiologists rated the patient’s satisfaction nearly the same as their own: 5.3 vs. 5.4. Regarding subgroups, female patients reported higher satisfaction than males (5.3 vs. 4.9), and outpatients were more satisfied than inpatients (5.5 vs. 4.7). Patients’ satisfaction was not as high as anticipated by the radiologists leading the briefing, who expected patients’ ratings of satisfaction to be better (overestimation of between 0.2 and 0.3 points).

Regarding performance, other studies have shown that physicians perceive their own performance as better than patients or colleagues do [42] and tend to overestimate their briefing performance and skills [43]. A significant discrepancy exists in the perception of communication between patients and physicians—often patients had negative impressions of the briefings, while physicians rated them as adequate or even excellent [36]. Tongue et al. [15] report that among a group of interviewed orthopedic surgeons, 75% perceived their own briefing style as “satisfactory,” while only 21% of patients felt the same.

Patient surveys consistently show a desire for better patient-physician communication [44] and—apart from physicians’ commitment to invest time—there are aspects of this which may be taught and learned. In 2002, Maguire and Pitceathly [45] proposed that in a patient-centered approach, the radiologist should avoid what they call “blocking behavior,” which consists of offering advice and reassurance before the main problems have been identified, switching the topic, and “jollying” patients along. In brief, radiologists could thus prevent patients from keeping important information to themselves, ensuring a safer examination procedure and patient satisfaction.

### Predictors of satisfaction

In this study, parameters influencing satisfaction, which may be actively worked on by radiologists, were of particular interest. To identify these parameters, we performed stepwise hierarchical regression to control for patients’ (e.g., demographic variables) and physicians’ characteristics (e.g., work experience or overestimation of patients’ satisfaction).

Research on parameters influencing patients’ satisfaction often explains less than 20% of the variance in patients’ satisfaction [46], indicating that essential predictors have yet to be identified. Our study explains a somewhat higher percentage, namely, 29% (see Table 3). In detail, patient and physician variables alone explained 21% of the variance, but communication variables accounted for an additional 8%. In contrast, other predictors may still be unidentified. Physicians’ characteristics explain a small amount, as mentioned by Jackson et al. [46]. In our study, physician characteristics explained only 1% of the variance in patient satisfaction (1%).

In contrast, patients’ expectations are independent predictors of satisfaction and may explain up to 10% of variance [47]. We did not study this parameter in particular because our study tried to identify those expectations related to briefings in a radiological setting. In our study, clarity of the briefing was the strongest predictor of patients’ satisfaction (see Table 3) and may be characterized as something patients expect the radiologists to deliver. As mentioned before, clarity was examined independently from the patients’ perception of patient-centeredness communication. Interestingly, in our study, patient-centeredness did not significantly predict patients’ satisfaction when controlled for other variables such as perceived clarity (see Table 3). This indicates that the patient’s value a clear communication more than the opportunity to ask questions or to have enough time to understand the briefing. This is in accordance with current literature, where clarity of the radiologist’s briefing is emphasized as especially necessary for the patient‘s understanding of the briefing [48]. Communicating radiation exposure, e.g., is often experienced as inadequate because, in its established implementation, a certain level of “numeracy” on the patient’s side is a prerequisite for proper comprehension. For better performance, authors such as Oesterling et al. [49] state clearly that quantitative data are “not always the most important consideration in reaching a decision” for a patient. Radiologists should stress qualitative aspects and thus help patients make a decision. Our results would support this approach—if patients had enough time to understand the doctor’s explanations if they confirmed understanding if they were allowed to ask questions and remembered the doctor’s name, they tended to be more satisfied than if two or more of these communication characteristics were not present.

Regarding patients’ characteristics, inpatient/outpatient status had the most substantial influence on satisfaction in our study. We found that outpatients were more satisfied than inpatients. The reason for this may be that outpatients will be more likely to experience just the radiology department during their visit, while inpatients interact with several departments or a variety of services. Other studies found older patients to be more satisfied than the younger patients (reviewed in [50]), a finding we could not confirm from our data. These findings were consistent with the results of other studies regarding the missing influence of gender [51–53].

The literature also suggests that patients’ satisfaction with a procedure is associated with its cost: the more expensive the treatment, the less satisfied patients are likely to be [54]. We did not compare briefings prior to the CT to briefings, e.g., prior to an intervention.

### Other factors not influencing patient satisfaction

Surprisingly, some patient-related factors did not influence patient satisfaction. Those patients with higher education were neither more nor less satisfied with the briefing. The actual and perceived length of the briefing did not influence satisfaction, nor did any prior informed consent briefings, patient age, or the degree of necessity a patient felt for informed consent briefings. Our results further show that the vast majority of our patients did not prepare for imaging in advance. Fewer than one-third of all patients used even a single source of information, and that single was usually the web.

We restricted our study of patient characteristics to more general demographic data, such as age, gender, etc. Because the literature suggests that patients’ satisfaction is influenced by health status, e.g., distress, anxiety, and depression (reviewed by [50]), future studies on communication variables predicting satisfaction with briefings prior to radiological examinations should control for those patient-related variables. Future studies should also include additional physician-related characteristics, such as overall qualification, qualification regarding doctor-patient communication, or a physician’s assessment of his own communication skills. Given the strong influence of the content of the briefing, which may be predefined, briefings by radiologists could also be compared to briefings given by a physician assistant.

## Limitations

Limitations of our study included the following. Radiologists were informed about the study, so that they might have performed the briefing more accurately/carefully than usual. This procedure, however, was inevitable because radiologists rated their interviews. Likewise, as the patients had to consent to participate in the study, they might have been more critical concerning the briefings. Patients’ satisfaction was assessed in a unidimensional approach—we did not differentiate between satisfaction with explanations and, e.g., satisfaction with inquiries into individual patients’ situations. Further investigations should focus on satisfaction with different aspects of the briefing situation, such as communication skills or behavior.

## Conclusion

Our study showed that overall satisfaction with briefings prior to contrast-enhanced CT is high and is predicted by patient-related factors such as gender and the setting of the examination but not by physician-related factors. The strongest predictor of a patient’s satisfaction was the perceived clarity of the briefing. This parameter depends on a well-developed scheme for the briefings, which could also be used by a physician assistant.

## Data Availability

The datasets used and/or analyzed during the current study are available from the corresponding author on reasonable request.

## Abbreviations

CT: Computed tomography
ESR: European Society of Radiology
